# Professor Ibrahim Saba Salti: A Titan of Arab Endocrinology

**DOI:** 10.7759/cureus.89463

**Published:** 2025-08-06

**Authors:** Mohamad Fleifel

**Affiliations:** 1 Endocrinology, Diabetes, and Metabolism, Ain Wazein Medical Village, Ain Wazein, LBN; 2 Endocrinology and Metabolism, Dr. Sulaiman Al Habib Medical Center-Al Ghadeer, Riyadh, SAU

**Keywords:** arab, aub, endocrinology and diabetes, historical vignette, lebanon

## Abstract

Professor Ibrahim Saba Salti was a Jerusalem native who became an Arab and an internationally respected legend in endocrinology. He was a man who was molded into a physician with a well-earned scientific pedigree in Lebanon. He got scholarly adopted by the American University of Beirut (AUB), from whom he adopted back to become one of its most iconic teachers and a revered professor of clinical medicine at its medical center (American University of Beirut Medical Center (AUBMC)). Prof. Salti's hard work was evident from a young age, from once holding a record of the youngest freshman to enter AUB to being the youngest medical graduate. From his work in establishing Lebanese, Arab, and international medical societies to having hundreds of scientific publications under his belt, Prof. Salti never failed to delicately and meticulously serve his patients in the field of endocrinology. He was a kind-hearted physician, scholar, and father figure who will never be matched again.

## Introduction and background

A man of few words and full dedication to practicing medicine in a compassionate and comprehensive way, in addition to teaching protégés the wonderful field of medicine in its finest details, he is Professor Ibrahim Saba Salti, an innate teacher, scholar, and physician. Prof. Salti's name has been synonymous with endocrinology in the Arab world for more than half a century [1]. His 50-plus-year career in the medical field inspired many physicians, scientists, and scholars to pursue their work in the name of medicine and science. Many excelled at the foundations that he laid down, and others marched on the path he paved. The list includes the likes of Dr. Baha Arafah and Prof. Sami Azar, who each have shone in their respective clinical and research fields. This article serves to shed light on some of Prof. Salti's life events and path to scholarly and scientific immortality.

## Review

A bittersweet yet fruitful journey

Professor Ibrahim Saba Salti, the grandson of a Christian Orthodox priest from Jerusalem, was born in 1941 in Palestine. Due to forced displacement in the mid-to-late 1940s, he became an expatriate and found a new home in Beirut, Lebanon [2]. Prof. Salti's life alternated between two sister Arab cities in Beirut and Amman, Jordan. His brilliance was evident from a young age. While attending school in Amman, he would partake in exams with students who were years ahead of him and still come out on top [3]. Despite his patriarch's wishes of choosing a business-related major, Prof. Salti had other plans, which shaped his future and the lives of many physicians to come after him.

Prof. Salti's academic excellence was distinct from his days as a university student, as he joined the American University of Beirut (AUB) in 1955. For a long time, Prof. Salti held the record for being the youngest individual to enter AUB as a freshman. He received his Doctor of Medicine (MD) degree, with distinction, in 1963 and completed his internal medicine residency in 1966, both at the American University Hospital (AUH, now AUB Medical Center (AUBMC)). He received his Royal College of Physicians of Canada endocrinology board certification from the University of Toronto in 1966. Prof. Salti's choice of endocrinology came about after he singlehandedly diagnosed his mother's pituitary disease. He also obtained a PhD in Medical Sciences from the Institute of Medical Sciences at the University of Toronto in 1970 [4]. It is said that he was offered the position of the dean of medicine at McMaster University, which he respectfully declined to continue his teaching and clinical practice back in the Arab region [3].

An embodiment of dedication

Prof. Salti returned to his foster home country of Lebanon and rejoined AUH as a full-time faculty member in the early 1970s. Apart from the nine-month sabbatical leave to Amman in the late 1970s, he has since remained with AUBMC and reached his professor rank in only two years after initially joining the institute. He established the first clinical endocrinology fellowship program in Lebanon and the Middle East, which has graduated many successful endocrinologists since its inception, who now practice across the world. Prof. Salti served as the head of the division for many years after its establishment, and the program continues to thrive as of the date of this article. The program has since expanded more to house both clinical and research arms, and it received its first Accreditation Council for Graduate Medical Education (ACGME) accreditation in 2024. Prof. Salti was successful in establishing the first endocrine radioimmunoassay in 1970 under the supervision of the endocrine division's then-chairperson, the late Dr. Najib Abu Haidar [5]. ​​The Endocrine COre REsearch (ECORE) laboratory served as a reference for all endocrine assays performed at the AUB until 2008. The ECORE laboratory successfully met the Centers for Disease Control and Prevention (CDC) Accuracy-based Monitoring Programs (CDC AMP) criteria for the years 2019-2022.​ As of the date of this article, the laboratory continues functioning and improving with its state-of-the-art instruments and devoted laboratory technologists [4,5]. Prof. Salti was appointed as deputy president of AUB from 1987 to 1993, which was one of the toughest times in Lebanon, given the ongoing civil war. Despite the waning turbulence of the war, AUB was the target of vandalism and destruction; however, Prof. Salti's calmness and decisiveness glued the institute together as his reign continued to lead AUB to a slow resurgence [4].

A local name beyond borders

Prof. Salti never forgot his home roots as he had always hoped to return to Palestine; however, this unfortunately never happened. His perseverance in establishing relations between medical faculties from sister Arab universities and forming international and regional scientific societies resulted in more success. Prof. Salti pioneered multiple medical societies and groups with other Arab comrades abroad. Along with Prof. Salman Abu Sitta, they formed the Canadian Arab Federation, and Prof. Salti was selected as its inaugural president [2]. He was among the first physicians to form the Lebanese Society for Endocrinology, Diabetes and Lipids (LSEDL) and was one of the endocrinologists who suggested a Pan-Arab Conference for worldwide endocrinologists in the late 1990s [2]. After that, he was appointed the president for the first ever Pan-Arab Congress for Endocrinology and Diabetes held in Beirut. The congress was another great feat with the participation of over 400 worldwide endocrinologists [2,4]. Prof. Salti also pioneered the formation of the Arab Medical Council for Medical Specialties in the 1980s, the Arab Society for the Development of Medical Sciences, and the Arab Society for Diabetes and Endocrinology in the late 1990s [2].

**Figure 1:**
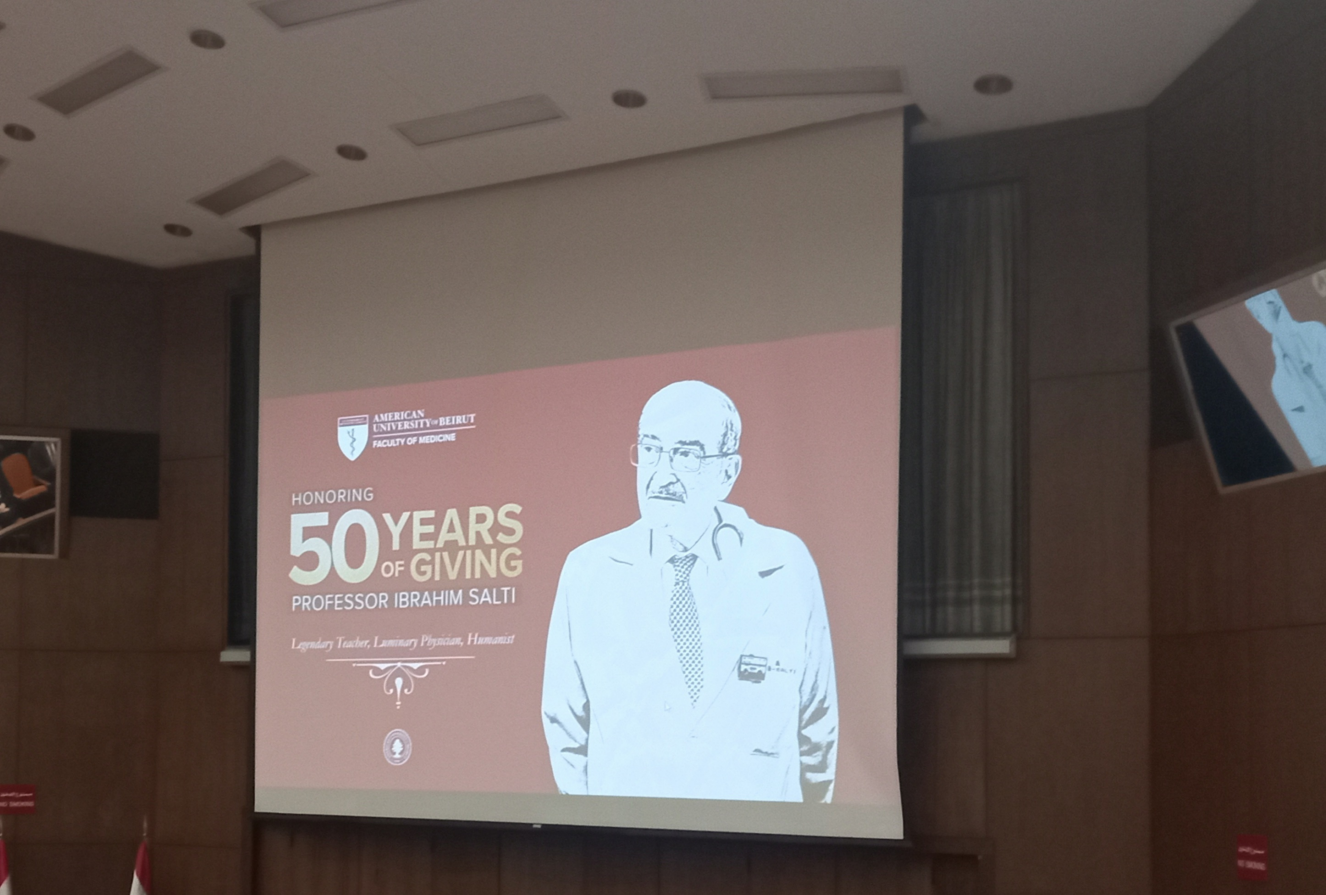
Ibrahim Saba Salti: a symbol of dedication Part of the "AUB honors Dr. Ibrahim Salti for over 50 years of dedication" ceremony. Image Credit: Dr. Mohamad Fleifel

Prof. Salti received many awards across his esteemed career, with one of them being the 2013 International Clinician Award from the American Association of Clinical Endocrinologists [6]. He was also awarded the prestigious Lebanese National Medal of Merit and the Jordanian Medal of Honor [7]. His most recent was an honorary ceremony held at AUB to celebrate his 50-plus years of dedication as a physician and teacher at the institute (Figure 1) [1,2]. Prof. Salti spearheaded and supervised hundreds of scientific publications that remain renowned to this day including the Epidemiology of Diabetes and Ramadan (EPIDAR) study as one of his finest research projects [4,8,9]. His other studies included investigating the acute and chronic dexamethasone usage on the parathyroid hormone (PTH)-induced rise in renal adenosine 3′,5′-cyclic phosphate, which showed no effect, and his take on continuing medical education in making it more accessible and effective to facilitate the learning of many healthcare professionals [10,11]. He was also among the first physicians to report the clinical experience of insulin pumps in Lebanon and the surrounding region [12]. Prof. Salti was also among the researchers who noted that type 1 diabetes patients receiving intensive insulin therapy had higher leptin levels compared to those receiving conventional insulin therapy [13].

A brief tribute

**Figure 2:**
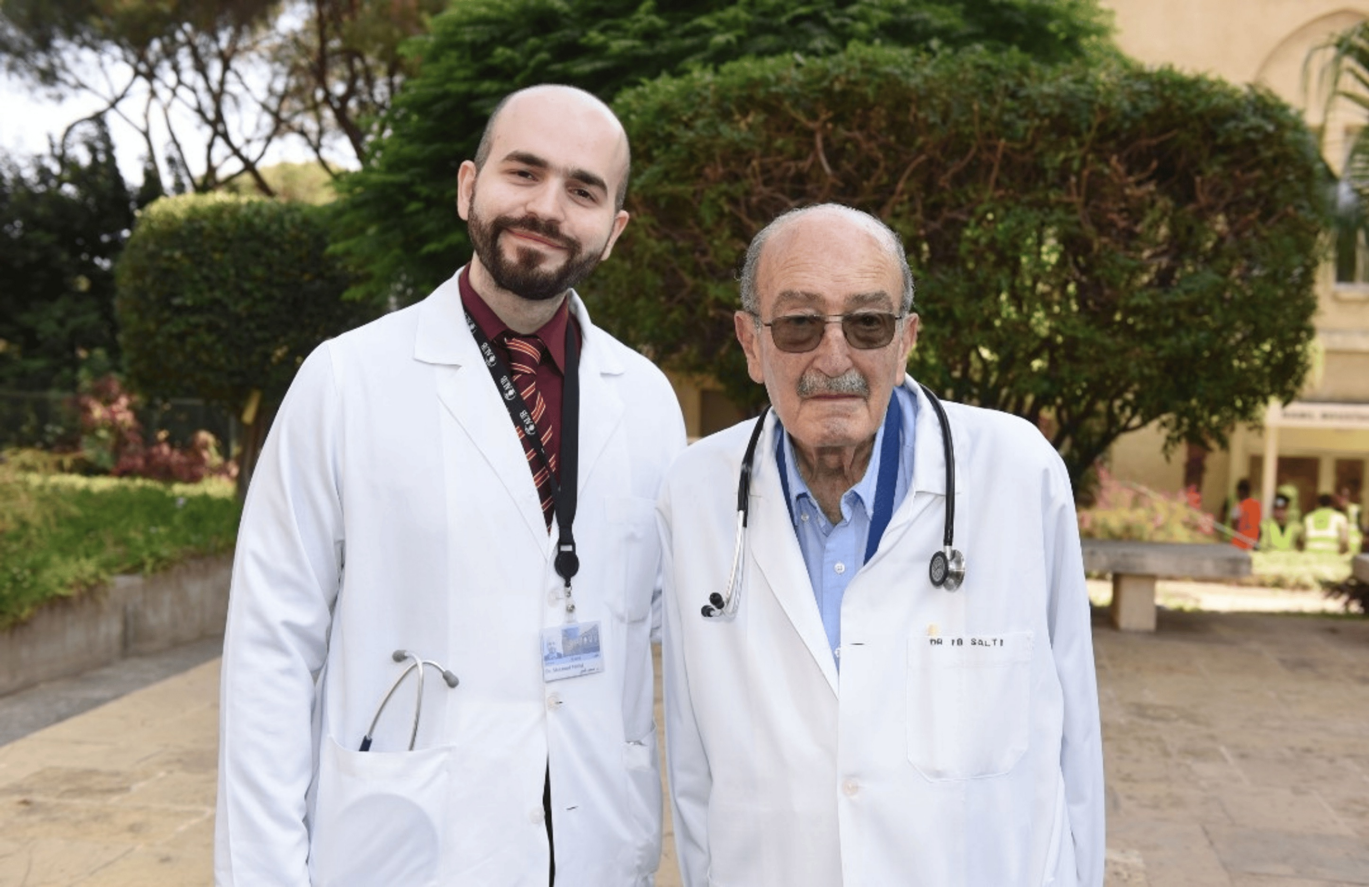
Ibrahim Saba Salti (right): esteemed leader. (Pictured next to the author of the article, Dr. Mohamad Fleifel) Image Credit: Dr. Mohamad Fleifel

It is an honor and a pleasure to document Prof. Salti's achievements and influence on Arab physicians and worldwide medicine in a medical journal. No matter the paragraphs, sentences, or words used, it remains difficult to describe the aura that Prof. Salti brings to the table even after his passing in September 2024. This article hopes to serve as a modest gesture to a giant of whom many, including myself (Figure 2), stood on his shoulders to reach where we are today.

## Conclusions

Prof. Salti was known to be an innovative and brilliant physician who was kind at heart. His revolutionary work in clinical practice, research, and education makes him stand out among the greats in the Arab medical field. He was a consummate influence on his colleagues and many current physicians who have built their medical programs and facilities based on the blueprint that he first laid out. Critically acclaimed by his patients, students, and colleagues, Prof. Salti reflected the kindness and goodwill found in medicine. Calm, collected, intelligent, meticulous in his job, and precise in his words, he is a historical treasure of Lebanese and Arab world medicine, being in the field of endocrinology and more. He remains unmatched and an untouched titan even after his passing. Prof. Salti is indeed an everlastingly shining gem that is hard to be duplicated.
